# Feeding and smoking habits as cumulative risk factors for early childhood caries in toddlers, after adjustment for several behavioral determinants: a retrospective study

**DOI:** 10.1186/1471-2431-14-45

**Published:** 2014-02-15

**Authors:** Alessandra Majorana, Maria Grazia Cagetti, Elena Bardellini, Francesca Amadori, Giulio Conti, Laura Strohmenger, Guglielmo Campus

**Affiliations:** 1 Dental School, Department of Pediatric Dentistry, University of Brescia, Brescia, Italy; 2 Department of Health Science, WHO Collaborating Center of Milan for Epidemiology and Community Dentistry, University of Milan, Milan, Italy; 3 IRCCS Ca’Granda University of Milan, Milan, Italy; 4 Department of Surgery, Microsurgery and Medicine Sciences – Dental School, University of Sassari, I-07100 Sassari, Italy

**Keywords:** Early childhood caries, Toddler, Feeding practice, Smoking exposure, Socio-Economic Status

## Abstract

**Background:**

Several maternal health determinants during the first period of life of the child, as feeding practice, smoking habit and socio-economic level, are involved in early childhood health problems, as caries development. The potential associations among early childhood caries, feeding practices, maternal and environmental smoking exposure, Socio-Economic Status (SES) and several behavioral determinants were investigated.

**Methods:**

Italian toddlers (n = 2395) aged 24–30 months were recruited and information on feeding practices, sweet dietary habit, maternal smoking habit, SES, and fluoride supplementation in the first year of life was obtained throughout a questionnaire administered to mothers. Caries lesions in toddlers were identified in visual/tactile examinations and classified using the International Caries Detection and Assessment System (ICDAS). Associations between toddlers’ caries data and mothers’ questionnaire data were assessed using chi-squared test. Ordinal logistic regression was used to analyze associations among caries severity level (ICDAS score), behavioral factors and SES (using mean housing price per square meter as a proxy).

**Results:**

Caries prevalence and severity levels were significantly lower in toddlers who were exclusively breastfed and those who received mixed feeding with a moderate–high breast milk component, compared with toddlers who received low mixed feeding and those exclusively fed with formula (p < 0.01). No moderate and high caries severity levels were observed in an exclusively breastfed children. High caries severity levels were significantly associated with sweet beverages (p < 0.04) and SES (p < 0.01). Toddlers whose mothers smoked five or more cigarettes/day during pregnancy showed a higher caries severity level (p < 0.01) respect to those whose mothers did not smoke. Environmental exposure to smoke during the first year of life was also significantly associated with caries severity (odds ratio =7.14, 95% confidence interval = 6.07-7.28). No association was observed between caries severity level and fluoride supplementation. More than 50% of toddlers belonging to families with a low SES, showed moderate or high severity caries levels (p < 0.01).

**Conclusions:**

Higher caries severity levels were observed in toddlers fed with infant formula and exposed to smoke during pregnancy living in area with a low mean housing price per square meter.

## Background

Breast milk is the ideal food for infants, providing all nutrients and antibodies that they require [1]. Feeding with human breast milk is considered to be the single most important preventive intervention for infant survival. In developing countries, exclusive breastfeeding for the first six months of life has been estimated to prevent 13% of deaths each year in children less than 5 years old [2]. Breastfeeding is defined as exclusive when no other supplement, such as water, juice, non-human milk, or food, excepting drugs, vitamins and minerals, is administered to the infant [1].

The association between socio-economic factors like maternal age, maternal education level, household income, mean cost of the housing in the area where the family lives and infant feeding practice are complex and may be even interrelated; in addition, the relations identified in bivariate analysis may not hold in multiple analysis and new association may be uncovered. Moreover, it is really tricky to capture the material and financial aspects of SES. An inverse association is also reported: the duration of breastfeeding was referred to be the highest among the mothers of the lower income group followed by mothers from the upper income groups [2]. Another study reports that mothers with a higher level of education started breastfeeding and more continued for the first 2 months after birth [3]. The literature on the determinants of breastfeeding has [3-5] consistently identified maternal smoking as predictor of lower breastfeeding rates. Babies whose mothers reported smoking during pregnancy were less likely to be breastfed [6,7]. Moreover, the development of caries in children may be associated with prenatal maternal smoking and postnatal environmental smoke exposure [8-11]. Maternal smoking during pregnancy appears to be a proxy for the mother’s unhealthy diet and poor oral hygiene practices [9-11].

Despite great efforts and achievements in oral health promotion, caries remains a major childhood health problem [12]. In Italy, as in the majority of industrialized countries, recent data have revealed that caries is distributed unevenly, with the highest burden evident in underprivileged groups [13,14]; this situation highlights the need for novel complementary strategies in caries prevention efforts.

The term early childhood caries (ECC) encompasses any form of caries occurring in infants, toddlers and preschool-aged children [12]. The pattern of caries in toddlers aged 12–30 months is specific. Current evidence suggests that the practice of nocturnal bottle-feeding with beverages containing sugar is the most important etiological factor in caries development [15]. Interactions among social, behavioral and microbiological factors, including several risk factors, also contribute to this process [13-18]. Epidemiological data focusing on ECC prevalence in toddlers are scarce [19]. An association between ECC and breastfeeding has been proposed, especially when breast milk is consumed *ad libitum*, in several daytime and nocturnal intakes, over a prolonged period [20]. Milk residues that accumulate in the mouth, promote caries development, especially during the night, when the salivary flow rate is reduced; however, the results of studies examining this association have been inconclusive [18,19].

Milk and milk products contain nutrients, such as calcium, phosphate, casein, and lipids, with potential anti-caries properties [21]. In Italy, the daily consumption of milk was associated with a lower prevalence of caries in schoolchildren with no fluoride supplementation and poor oral hygiene [22]. However, studies of the association between dairy product intake and dental caries in young children have been rare, and the results have been inconsistent [21,22].

The aim of this retrospective study was to investigate the potential association between feeding practices, maternal and environmental smoking exposure and SES as risk factors for caries development in toddlers aged 24–30 months.

## Methods

### Study design and participants

The study was performed in the city of Brescia (Italy) and was approved by the Ethics Committee of Brescia Hospital (no. 298/2007). The metropolitan area of Brescia includes about 1,210,000 inhabitants [http://www.demo.istat.it], and the fluoride content in tap water is low (0.12–0.05 mg/l) [http://www.a2acicloidrico.eu/home/cms/idrico/].

Mothers attending the two obstetric wards of Brescia Hospital were enrolled between May 2008 and April 2009 (total number of births 3523). All mothers who gave birth to a healthy child at full term (≥37 weeks of pregnancy) with a birth weight ≥ 2500 g were invited to participate in the study. Exclusion criteria were maternal diseases that prevented breastfeeding, twin births and congenital oral cavity malformation (*e.g*., cleft palate) in children (number of eligible children 2623). An information leaflet explaining the aim of the study and requesting consent to participate was provided to the mothers. Only mothers who provided written consent (n = 2610) were enrolled.

**Figure 1:**
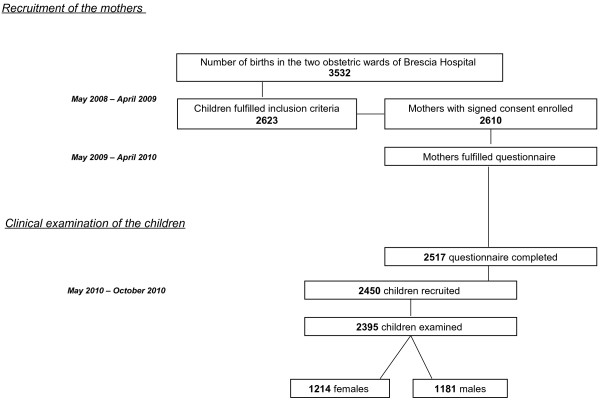
Flow chart of the study design.

Between May and November 2010, the children were recruited; the appropriate size of the toddler sample was calculated on the basis of a previous study of caries prevalence in Italian preschool children [5,12,14]. The previous sample size was increased by 15% (to 2410 subjects) to ensure an optimal level of precision (5%), given the possible effects of caries prevalence reduction and non-response. A total of 2450 subjects aged 24–30 months were recruited and 2395 (1214 females [50.69%], 1181 males [49.31%]) were examined. The flow chart of the study design is shown in Figure 1.

### Questionnaire

Mothers were contacted by e-mail, and/or phone at six, nine and twelve months after delivery and asked to complete a self-administered, highly structured questionnaire with closed questions regarding feeding practices, infant’s sweet dietary habit, smoking habit during pregnancy, environmental exposure of the infant to smoke, and fluoride supplementation during pregnancy and in the infant’s first year of life. The questionnaire was standardized and previously used in a National Pathfinder study showing a high reliability and validity (Cronbach’s alpha = 0.92) [13]. In case of non-receipt of the questionnaire, mothers were contacted by telephone or email on a weekly basis up to three calls; in addition unclear or incomplete answers were clarified through telephone communication.

Feeding practices were classified using cut-off points for the percentages of breast milk and formula administered to the infant at each meal. In the questionnaire was asked to the mothers every six weeks to report for three consecutive meals how many grams of each type of milk was given to the baby. A mean of each type of milk in the three meals was done and the values were expressed in percentages [23]. The cut-off points were: exclusive breastfeeding with 100% breast milk for 6 months, moderate–high mixed feeding with 58–99% breast milk, low mixed feeding with 1–57% breast milk, and exclusive use of formula (0% breast milk) [23]. Breastfeeding was also monitored at 9 and 12 months of age. Sweet dietary habit was investigated by inquiring about the administration of sweet beverages other than milk (*e.g.*, juice or other beverages rich in fermentable carbohydrates) at 6 and 12 months of the infant’s life. Maternal smoking during pregnancy was considered positive when the mother reported smoking more than five cigarettes/day. Environmental exposure to smoke was considered positive when more than five cigarettes/day were smoked at home. Prenatal fluoride supplementation (tablet/lozenge) was classified into three categories: no fluoride supplementation, supplementation with ≥1 mg/day for ≤12 weeks of pregnancy, and supplementation with ≥1 mg/day for more than 12 weeks of pregnancy [24]. Postnatal fluoride supplementation (drops) was measured by asking the mother about the use of fluoride supplementation at 6 and 12 months of the infant’s life.

The mean housing price per square meter in the area where the mothers live during pregnancy was used as a proxy of SES [25,26].

### Clinical examination

At the toddlers’ ages of 24–30 months, mothers were contacted by phone or e-mail and asked to bring their children to the Department of Pediatric Dentistry of the University of Brescia for clinical screening. Two calibrated examiners (EB, FA) performed dental screenings using a dental unit. Intra- and inter-examiner reliability was assessed before the beginning of the survey by examining and re-examining (after 72 h) sixty-five subsequent study participants. Inter-examiner reliability was evaluated using fixed-effects analysis of variance in comparison with benchmark values (GC). Intra-examiner reproducibility was assessed as the percentage of agreement using Cohen’s kappa statistic [27]. Good inter-examiner reliability was found for the collapsed ICDAS codes 1–3, 4 and 5–6, with no significant difference from benchmark values (p = 0.21) and a low mean square of error (0.44). Intra-examiner reliability was also high Cohen’s Kappa statistic = 0.84.

Just before dental examinations, mothers brushed toddlers’ teeth. The presence of carious lesions was assessed using a plain mirror and a World Health Organization periodontal probe, under optimal artificial lighting. Carious lesions were evaluated using the International Caries Detection and Assessment System (ICDAS) II criteria during visual and tactile examination; no radiographs were taken [28,29]. Following the ICDAS, decayed surfaces were coded as 1 when a first visual change in enamel, seen only after prolonged air drying or restricted to within the confines of a pit or fissure (including non-cavitated and cavitated lesions), was present; as 2 when a distinct visual change in enamel was detected; as 3 when localized enamel breakdown (with no clinical visual sign of dentinal involvement) was observed; as 4 when a underlying dark shadow from dentin was identified; as 5 when a distinct cavity with visible dentin was seen; and as 6 when an extensive, distinct cavity with visible dentin was discovered. Subjects were categorized according to maximum ICDAS score as follows: low caries severity level (1–3) referred to caries involving only the enamel with no evidence of dentine involved, moderate caries severity level (4) referred to caries involving enamel and dentine, and high caries severity level (5–6) referred to cavitated caries lesions.

### Data analysis

Data were entered into a database (Excel 2010; Microsoft Corporation, Redmond, WA, USA). Statistical analyses were performed using Stata® 10.0 software (http://www.stata.com). Responses to questionnaire items were treated as categorical or ordinal variables. The mean housing price per square meter in the area where the mother lives during pregnancy [30] was used as proxy of SES. This parameter was chosen because in Italy the majority of families (about 80%) live in their own home. The city of Brescia was divided into three different areas on the basis of the property price: city center with houses at a mean price of Euro 2.750 per square meter; semi-central area with houses at a mean price of Euro 2.140 square meter, and suburbs area at a mean price of Euro 1.780 per square meter (http://www.immobiliare.it/prezzi-mq/Lombardia/Brescia.html).

Associations between toddlers’ caries data, gender and mothers’ questionnaire data were assessed using chi-squared test. Ordinal logistic regression was used to analyze associations among caries severity level (ICDAS score), feeding practices, Socio-Economic Status and behavioral factors. The Akaike information criterion (AIC) was used to measure the goodness of fit of the statistical model. Multicollinearity might sometimes cause problems with regression results. This problem was solved using the DFBETA command in Stata, dropping the information that have too much influence on the regression line. Anyway, after the data elaboration, no statistical significant multicollinearty was observed and so it was decided to report findings without outliers [31]. Statistical significance was set at α = 0.05.

## Results

Data from 2517 questionnaires completed by mothers and 2395 dental examinations of toddlers were included in the analyses. Drop-out rates were low, as only 93 (3.56%) mothers submitted incomplete questionnaires and 122 (4.85%) toddlers were not present at the time of clinical examination or were excluded from the analyses (Figure 1). The main reason for study drop-out was relocation of the family outside of the community (n = 54 [2.15%] mothers); in addition, 16 (0.64%) mothers did not reply to the request to bring toddlers for examination, 30 (1.19%) toddlers were absent on the examination day, and 22 (0.87%) refused to undergo the examination.

Caries was present (ICDAS score ≠ 0) in 80.84% of toddlers; 48.60% had low caries severity level, 27.52% had moderate caries severity level, and 4.30% had high caries severity. Non-cavitated carious lesions were recorded most frequently (Table 1). The severity of caries did not differ significantly by gender (χ^2^_(3)_ = 3.37, p = 0.34; Table 1). ICDAS scores were significantly lower in children who received higher proportions of breast milk (exclusive breastfeeding, moderate–high mixed feeding) than in those who received lower proportions (low mixed feeding, exclusively infant formula) at 6 months of age (p < 0.01; Table 2). Mothers reported the continuation of exclusive breastfeeding at 9 months in 34.5% (n = 203) of toddlers who were exclusively breastfed at 6 months. At 12 months, only nine toddlers were still partly breastfed; ICDAS scores were 1–3 for all of these subjects. Different feeding practices were significantly associated with ICDAS scores (odds ratio [OR] = 6.75, 95% confidence interval [CI] = 6.00-7.58); moderate and high caries severity levels were not observed in subjects who were exclusively breastfed, whereas high caries severity level was predominant in children fed with formula (low mixed feeding, 58.43%; exclusively formula, 85.50%). The goodness of fit (AIC criterion) was 3920.29.

**Table 1:** Sample distribution according to gender and severity of caries

**Caries severity**	**Male n (%)**	**Female n (%)**	**Total n (%)**
No caries	227 (9.47)	232 (9.69)	459 (19.16)
Low (ICDAS 1–3)	580 (24.22)	584 (24.38)	1164 (48.60)
Moderate (ICDAS 4)	328 (13.69)	331 (13.82)	659 (27.52)
High (ICDAS 5, 6)	46 (1.92)	67 (2.81)	113 (4.72)

*Male vs. female: χ*^*2*^_*(3)*_ *= 3.37, p = 0.34.*

ICDAS, International Caries Detection and Assessment System.

**Table 2:** Distribution of caries severity according to behavioral and environmental factors

	Caries severity (ICDAS score)^*^					**Ordered logistic regression (n = 2395)**			
	No caries	Low (1–3)	Moderate (4)	High (5, 6)	Total	Log likelihood	p	OR (SE)	95% CI
**Feeding practice** (first 6 months)						-1956.14	<0.01	6.75 (0.40)	6.00–7.58
Exclusive breastfeeding	240 (40.82)	348 (59.18)	0 (0)	0 (0)	588 (24.55)				
Moderate–high mixed feeding	172 (23.40)	563 (76.60)	0 (0)	0 (0)	735 (30.69)				
Low mixed feeding	42 (7.86)	180 (33.71)	311 (58.24)	1 (0.19)	534 (22.30)				
Exclusively artificial formula	5 (0.93)	73 (13.57)	348 (64.68)	112 (20.82)	538 (22.46)				
**Sweet dietary habit**						-2767.84	0.04	1.18 (0.10)	0.99–1.40
≤1/day	366 (21.11)	814 (46.94)	486 (28.03)	68 (3.92)	1734 (72.40)				
≥2/day	93 (14.97)	360 (54.66)	173 (26.17)	35 (5.30)	661 (27.60)				
**Smoking habit**						-1873.25	<0.01	7.14 (0.37)	6.07–8.28
No smoking	397 (20.74)	948 (49.53)	490 (25.60)	79 (4.13)	1914 (79.92)				
During pregnancy	25 (15.53)	53 (32.92)	62 (38.51)	21 (13.04)	161 (6.72)				
Environmental exposure	37 (11.56)	163 (50.94)	107 (33.44)	13 (4.06)	320 (13.36)				
**Mean housing cost (SES)**						-2414.70	<0.01	0.21 (0.21)	0.18–0.24
Low	23 (3.27)	241 (34.23)	359 (50.99)	81 (50.99)	704 (29.39)				
Moderate	130 (12.06)	717 (66.51)	209 (19.39)	22 (2.04)	1078 (45.02)				
High	306 (49.92)	216 (35.24)	91 (14.84)	0 (0)	613 (25.59)				

*N (%); percentages are calculated within each column.

ICDAS, International Caries Detection and Assessment System; OR, odds ratio; SE, standard error; CI, confidence interval.

The frequency of sweet beverage feeding in the first 6 months was very low (3.05%), but 661 (27.60%) mothers reported giving their children sweet beverages more than once per day at 12 months of age. A significant association (p < 0.04) was found between ICDAS score and the provision of two or more sweet beverages a day (OR = 1.18, 95% CI = 0.99-1.40; Table 2). A high sweet beverages intake by the children was also highly statistically significant associated to smoking habit (χ^2^_2_ = 736.36 p < 0.01) (*data not in table*).

Children whose mothers reported smoking five or more cigarettes/day during pregnancy showed a higher risk for the development of caries (p < 0.01). Smoking habits (maternal during pregnancy, environmental exposure) were significantly associated with ICDAS scores (OR = 7.14, 95% CI = 6.07-8.28; Table 2). No child with high caries severity levels belonged to a family living in high-cost house, whereas more than 50% of toddlers belonging to mothers/families living in low-cost house had moderate or a high caries severity levels (p < 0.01).

Overall, 43.21% (n = 1127) of mothers reported the use of fluoride supplementation during pregnancy; the majority (n = 960) of these mothers reported using supplementation for less than 12 weeks of pregnancy. Prenatal fluoride supplementation was not associated with ICDAS score; few (3.7%) mothers reported the postnatal use of fluoride at 6 months, so this variable was excluded from the analysis (*data not shown*).

The estimates related to caries severity according to mean housing cost, feeding practice and smoking habit are displayed in Table 3 and in Table 4 without outliers. All the models were statistically significant (p < 0.01). Results displayed in Table 4 do not differentiate statistically significant from results displayed in Table 3. Feeding practice was the main risk factor associated to caries severity, followed by smoking habit and SES. The goodness of fit (AIC criterion) was 4040.70.

**Table 3:** Ordinal logistic regression estimation

***Number of observation = 2395 Caries severity***	***Log likelihood = -2785.29 OR (SE)***	***χ***^***2***^ ***= 2431.62 p < 0.01 *** **p-value (95% CI)**
*Mean housing cost (SES)*	0.34 (0.02)	<0.01 (0.30 – 0.38)
*Feeding practice*	7.57 (0.44)	<0.01 (6.77 – 8.48)
*Number of observation = 2395*	*Log likelihood = -3655.37*	*χ*^*2*^ *= 691.47 p < 0.01*
**Caries severity**	**OR (SE)**	**p-value (95% CI)**
*Mean housing cost (SES)*	0.24 (0.01)	<0.01 (0.22 – 0.27)
*Smoking habit*	1.66 (0.15)	<0.01 (1.38 – 1.99)
*Number of observation = 2395*	*Log likelihood = -2772.91*	*χ*^*2*^ *= 2456.38 p < 0.01*
**Caries severity**	**OR (SE)**	**p-value (95% CI)**
*Mean housing cost (SES)*	0.34 (0.02)	<0.01 (0.30 – 0.38)
*Feeding practice*	7.58 (0.44)	<0.01 (6.77 – 8.48)
*Smoking habit*	1.62 (0.16)	<0.01 (1.34 – 1.96)
*Number of observation = 2395*	*Log likelihood = -2940.91*	*χ*^*2*^ *= 2120.38 p < 0.01*
**Caries severity**	**OR (SE)**	**p-value (95% CI)**
*Feeding practice*	8.31 (0.47)	<0.01 (7.44 – 9.29)
*Smoking habit*	1.61 (0.15)	<0.01 (1.34 – 1.94)

Caries severity according to feeding practice smoking habit and mean housing cost (SES).

**Table 4:** Ordinal logistic regression estimation without outliers

***Number of observation = 2395 Caries severity***	***Log likelihood = -3902.84 OR (SE)***	***χ***^***2***^ ***= 196.52 p < 0.01 *** **p-value (95% CI)**
*Mean housing cost*	21.72 (44.74)	0.13 (0.38 – 1230.24)
*Feeding practice*	1.54^e-12^ (3.51^e-12^)	<0.01 (1.76^e-14^ – 1.35^e-10^)
*Number of observation = 2395*	*Log likelihood = -3974.17*	*χ*^*2*^ *= 53.86 p < 0.01*
**Caries severity**	**OR (SE)**	**p-value (95% CI)**
*Mean housing cost*	188162.9 (349924.1)	<0.01 (4915.47 – 7202820)
*Smoking habit*	116.08 (233.24)	0.02 (2.26 - 5957.48)
*Number of observation = 2395*	*Log likelihood = -3848.88*	*χ*^*2*^ *= 304.45 p < 0.01*
**Caries severity**	**OR (SE)**	**p-value (95% CI)**
*Mean housing cost*	0.10 (0.01)	0.06 (0.00 – 1.01)
*Feeding practice*	7.36^e-19^ (2.02^e-18^)	<0.01 (3.41^e-21^ – 1.59^e-16^)
*Smoking habit*	4.25^e+10^ (1.033^e+11^)	<0.01 (3.77^e+8^ - 4.79^e+12^)
*Number of observation = 2395*	*Log likelihood = -3852.00*	*χ*^*2*^ *= 298.20 p < 0.01*
**Caries severity**	**OR (SE)**	**p-value (95% CI)**
*Feeding practice*	2.09^e-17^ (4.99^e-17^)	<0.01 (1.95^e-19^ – 2.25^e-15^)
*Smoking habit*	5.40^e+9^ (1.23^e+10^)	<0.01 (6.129^e+7^ - 4.63^e+11^)

Caries severity according to mean housing cost feeding practice and smoking habit.

## Discussion

This study aimed to elucidate the potential associations among feeding practices, maternal and environmental smoking exposure and SES as risk factors for caries development in toddlers aged 24–30 months.

The health benefits of breastfeeding are widely recognized [1]; the majority of children in the present sample had been exclusively or partly breastfed, as reported in several northern European countries [32]. In this study, early-life feeding practices were significantly associated with dental caries severity (measured using ICDAS II criteria). Caries scores in all ICDAS categories were recorded in all four categories of feeding practice, but the highest caries severity levels were more likely to occur in toddlers who had received lower percentages of breast milk (low mixed feeding or exclusively formula). A diet rich in non-milk sugars has been considered to be cariogenic for infants and toddlers [32]. In addition, the early introduction of sugary foods and beverages is known to lead to the establishment of a habit that persists over time [33]. In contrast, breastfeeding has also been associated with an increased prevalence of ECC, although researchers’ opinions on this matter have differed [8,14-18]. The evidence reported is quite inconclusive and the association has been found only in children receiving prolonged and nocturnal breastfeeding. Moreover, a systematic review determined that no scientific evidence supports the cariogenic capacity of human milk [19]. Contradictory results among studies may be explained primarily by methodological disparities, such as the use of different cut-off points for breastfeeding [19,20].

In this study, caries in toddlers was associated with maternal smoking during pregnancy, which might be considered a proxy for unhealthy dietary and oral hygiene habits [9,11]. Environmental exposure to smoke was also significantly associated with high caries levels even it is probably that smoking during pregnancy is likely to be underreported, which was slightly the case in the population object of the study. Toddlers exposed to smoke were also less likely to be breastfed. Furthermore, the highest caries figures were observed in children fed with infant formula and exposed to smoke (maternal and environmental). To our knowledge, no study of the effects of smoking exposure and feeding practice on the risk ECC development has been reported previously. Caries development in the primary dentition, especially in very young children, is related to various social, demographic, and behavioral factors, including SES [34]; differing aspects of SES may be associated with knowledge, attitudes, experiences, and beliefs leading a woman to a particular infant feeding choice [35].

Fluoride supplementation during pregnancy was not found to be significantly associated with toddlers’ caries scores. Fluoride is recognized as a major factor in caries decline in many countries over the last several decades [12]. Dental hard tissue acquires fluoride systemically and topically; fluoride-rich enamel resists acid produced by cariogenic bacteria much better than does enamel lacking fluoride [36]. Studies of the effect of fluoride exposure during the prenatal period have been rare, although it has been shown to result in no additional measurable uptake by dental tissues other than that attributable to postnatal fluoride alone [36-38]. In the present sample, prenatal fluoride exposure appeared to have no effect on caries risk profiles. Furthermore, the effect of postnatal fluoride administration (drops) to toddlers was negligible, and this variable was consequently not included in the multivariate analysis. The Italian National Guidelines on caries prevention in childhood, released by the Italian Ministry of Health in 2008 and revised in 2013, leave to the pediatricians the choice to administer fluoride using a systemic or topical procedure and this might be related to the negligible frequency of fluoride supplement administered to the toddlers enrolled in this study [39].

This study has some limitations. Feeding habits were investigated during the first year of infants’ lives using a self-administered questionnaire distributed to mothers to reduce the possibility of recall bias. However, the effects of variables may have been attenuated, rather than increased, by this method of data collection [37]. Secondly, fluoride intake from toothpaste was not considered. Third, the use of the mean housing price per square meter as a proxy of SES might be criticized. For example, low-income families living in high-income neighborhoods have better access to health care than do families with similar incomes living in low-income neighborhoods [39]. However, given that more than 80% of Italian citizens own houses [http://www.demo.istat.it], area-based measures can be used to investigate differences in SES [40]. Misclassification or underreporting of the determinants of breastfeeding, and in particular smoking status, may have led to residual confounding resulting in a lack of an explanation for the association observed between SES and breastfeeding. Moreover, only one aspect of the socioeconomic variables was measured, so it was not possible to rule out additional unmeasured effects of SES on caries data or on the other caries risk factors. The results of the present study can be generalized for populations with similar levels of risk factors exposure.

## Conclusion

In conclusion, a positive association among ECC, feeding practice, smoking habits in the first six months of life was verified. Furthermore the role of other important risk factors was enlightened, like SES etc. No-exclusively breastfeeding showed to increase caries severity and it is very important that pediatricians are aware of these associations to direct toddlers at risk to the pediatric dentists.

## Competing interests

All authors declare that they have no potential conflict of interest.

## Authors’ contributions

AM and LS conceived of the study and participated in its design; MCG participated in the design of the study and drafting of the manuscript; EB and FA collected data; GC organized dental examination appointments and administered and collected questionnaires; and GCampus performed the statistical analysis and was involved in the drafting of the manuscript. All authors read and approved the final manuscript.

## Pre-publication history

The pre-publication history for this paper can be accessed here:

http://www.biomedcentral.com/1471-2431/14/45/prepub
